# The Importance of Lean Body Mass for the Rate of Force Development in Taekwondo Athletes and Track and Field Throwers

**DOI:** 10.3390/jfmk3030043

**Published:** 2018-08-10

**Authors:** Angeliki Kavvoura, Nikolaos Zaras, Angeliki-Nikoletta Stasinaki, Giannis Arnaoutis, Spyridon Methenitis, Gerasimos Terzis

**Affiliations:** 1 Sports Performance Laboratory, School of Physical Education & Sports Science, National and Kapodistrian University of Athens, Ethnikis Antistassis 41, Daphne, 17237 Athens, Greece; 2 Laboratory of Dietetics, Department of Dietetics and Nutritional Science, Harokopion University of Athens, El.Venizelou Str, 70, Kalithea, 17671 Athens, Greece

**Keywords:** combat sports, muscle power, body composition, muscle architecture

## Abstract

The rate of force development (RFD) is vital for power athletes. Lean body mass (LBM) is considered to be an essential contributor to RFD, nevertheless high RFD may be achieved by athletes with either high or low LBM. The aim of the study was to describe the relationship between lower-body LBM and RFD, and to compare RFD in taekwondo athletes and track and field (T&F) throwers, the latter having higher LBM when compared to taekwondo athletes. Nine taekwondo athletes and nine T&F throwers were evaluated for countermovement jumping, isometric leg press and leg extension RFD, vastus lateralis (VL), and medial gastrocnemius muscle architecture and body composition. Lower body LBM was correlated with RFD 0–250 ms (*r* = 0.81, *p* = 0.016). Taekwondo athletes had lower LBM and jumping power per LBM. RFD was similar between groups at 30–50 ms, but higher for throwers at 80–250 ms. RFD adjusted for VL thickness was higher in taekwondo athletes at 30 ms, but higher in throwers at 200–250 ms. These results suggest that lower body LBM is correlated with RFD in power trained athletes. RFD adjusted for VL thickness might be more relevant to evaluate in power athletes with low LBM, while late RFD might be more relevant to evaluate in athletes with higher LBM.

## 1. Introduction

Explosive strength is of great importance for performance in several power-demanding sports, such as sprints, long and high jumps, athletic throws, and martial arts. Explosive strength may be evaluated with the rate of force development (RFD; i.e., the slope of the tangent line of the force/torque-time curve during an explosive muscle contraction [1,2]), which has been linked with explosive sports performance. Indeed, lower body isometric leg press RFD at 100, 150, 200, and 250 ms from the onset of contraction, is associated with performance in track and field throwers, where the time for the final delivery of the implement is between 150–250 ms [3]. Taekwondo is also considered to be a powerful sport. For example, one of the most effective kicks in taekwondo, the roundhouse kick to the head, is developed between in approximately 250 ms (time from the instant that the striking foot leaves the floor until it reaches the objective and achieves the maximum impact force) [4,5,6]. This kick is characterized as a high velocity unloaded movement, in contrast to track and field throws where a relatively heavy external throwing implement is used. Thus, it might be postulated that RFD might be equally important for taekwondo athletes as for throwers. However, RFD in taekwondo athletes has not been described before.

RFD is influenced by different factors at early (<100 ms) and late phases (>100 ms) from the onset of muscle contraction [2,7,8]. The early phase of RFD is thought to be determined mainly by the neural drive to the muscle, as well as the muscle fiber type composition [2,7,8,9]. Late phase RFD is largely influenced by maximal strength and most likely muscle mass, as suggested by studies reporting parallel changes in maximal muscle strength and contractile RFD 150–250 ms from the onset of contraction [1,2,7,8,10,11,12,13]. Nevertheless, little is known about the correlation between muscle mass and RFD in power athletes. Recently, a close relationship was reported between isometric leg press late RFD (>100 ms) and lean body mass in young aged track and field throwers, while early RFD (<100 ms) was not related with their lean body mass [14]. Taekwondo athletes are expected to have lower lean body mass when compared to track and field throwers [15], although direct evidence is lacking on this issue. This may suggest a lower late RFD and lower maximum isometric force (MIF) and/or torque (MIT) in taekwondo athletes as compared to that of throwers, although this remains to be evaluated.

Muscle architectural characteristics, fascicle length, pennation angle, and muscle thickness, may also influence RDF [2,14,16]. Previous studies indicate that pennation angle and muscle thickness seems to affect late RFD [2,14,17,18]. Fascicle length is thought to reflect the number of sarcomeres in series in a muscle and the increase in this number has been suggested to contribute to higher shortening velocity and muscle power [19]. Longer fascicles are typically found in agonist muscles of faster sprinters [20,21,22], while studies in humans have reported longitudinal muscle growth in response to power training [23,24,25,26,27]. Recently, a correlation was found between vastus lateralis fascicle length and late RFD (100–250 ms) in young aged track and field throwers [14]. In addition, previous study indicates that in experienced power trained athletes, vastus lateralis fascicle length have stronger contribution/effect on sprinting, jumping, and throwing performance as compared to pennation angle and muscle thickness [28]. However, the relationship between muscle architecture and early and late RFD in athletes is still debatable, while there is no such information for taekwondo athletes.

The aim of the study was to describe the relationship between lower-body lean mass and RFD and to compare lower-body RFD in taekwondo athletes and track and field throwers, the latter having higher lean body mass when compared to taekwondo athletes. For this purpose, maximal isometric leg press and leg extension RFD curves were recorded from taekwondo athletes and track and field throwers of national and international level. It was hypothesized that (1) lower body lean mass would be associated with RFD in both athletic populations, but also (2) that they would differ in early and late RFD characteristics. Muscle architecture was also evaluated to provide further insight into the muscle morphology origin of the anticipated differences in RFD between the two athletic groups.

## 2. Materials and Methods

### 2.1. Experimental Design

All of the measurements were performed within 20–30 days before the national championship for each athletic group. Athletes visited the laboratory on four different occasions. On day 1, they gave their written consent to participate in the study, and their lower-extremity dominance and anthropometry were evaluated. Familiarization with the countermovement jump (CMJ), isometric leg press and leg extension RFD and maximal isometric force/torque were also performed. On day 2, three days later, athletes visited the laboratory for muscle architecture evaluation and another testing familiarization. On day 3, three days later, they performed the testing session. On day 4, three to four days after the testing session, body composition analysis was performed (see description below). All of the procedures were approved by the local university ethics committee.

### 2.2. Participants

Nine male taekwondo athletes (age 21.22 ± 2.90 years, range 19–28 years; body height 174.8 ± 0.05 cm; body mass 67.6 ± 8.3 kg; competition experience = 7.7 ± 4.6 years) and nine male track and field throwers (age 23.66 ± 5.04 years, range 19–34 years; body height 183.3 ± 0.05 cm; body mass 99.3 ± 9.9 kg; competition experience = 7.7 ± 3.9 years) gave their written consent to participate in the study after being informed about the experimental procedures as well as the risks and benefits of this research. Four of the taekwondo athletes were champions in the previous three years within separate weight classes. The rest of the taekwondo athletes placed second or third in the national championship or the national cup competition in the recent years. All of the taekwondo athletes participated in international competitions during the previous two years. The group of track and field throwers included two javelin throwers (mean performance: 79.8 ± 1.8 m), four shot putters (mean performance: 15.6 ± 0.9 m), and three hammer throwers (mean performance: 70.5 ± 4.3 m). The javelin throwers were the national champions in junior and senior categories, respectively, and were also among the best eight throwers of the European championship in the respective age categories, during the previous two years. Two of the shot putters placed first and second of the last years’ national outdoor championship while the others ranked 9th and 11th. One of the hammer throwers was among the eight best of the European championship under 23 years, while the other two placed 3rd and 4th at the national championship, during the previous year. After detailed oral and written description of the procedures, they gave their written consent to participate in the study. All of the procedures were approved by the School of Physical Education & Sports Science, National and Kapodistrian University of Athens Ethics Committee (registration number: 1039/14/02/2018).

### 2.3. Training Background

Taekwondo athletes were following a training program that was designed to develop technical skills and increase both their anaerobic and aerobic capacity to face the competition needs as recently described [15]. Resistance training for the athletes in this study was performed 2–3 sessions per week for the previous 4–5 years, including low resistance loads (60–70% of 1RM) with maximum velocity movements using mainly structural exercises (e.g., squat, bench press, leg extension). Body mass explosive exercises were also included in 3–4 sessions per week (e.g., jumps, push ups). On the other hand, throwers performed resistance training 3–4 training sessions per week during the previous 6–8 years using high resistance loads (80–95% of 1RM) with structural exercises (squats, bench press, shoulder press, etc.). Olympic weightlifting exercises performed with high movement velocity were also included [14]. Plyometrics were also performed in 2–3 sessions each week using the body mass as external loading. 

### 2.4. Testing Procedures

#### 2.4.1. Countermovement Jumps

Countermovement jumps were performed on a force platform (Applied Measurements Ltd. Co., Aldermaston, Berkshire, UK, WP 800–1000 kg weighting platform, 80 × 80 cm, sampling frequency 1 kHz) with hands on hips with elbows pointing out. Briefly, after a short warm up on a stationary bicycle, athletes performed three sub-maximal CMJ’s with progressively higher intensity. Then, athletes performed three CMJ’s with maximum intensity, instructed to jump as high as possible. Two-minute rest was allowed between attempts. Data from the force platform were recorded and analysed (Kyowa sensor interface PCD-320A; Kyowa Electronic Instruments Co., Ltd., Chofu, Tokyo, Japan) to calculate the maximum vertical jump height, the power output, the velocity, and the average rate of force development [ARFD (N·s^−1^) = (Max force − Body weight) × [(Time of maximum force − Time at which force reach the body weight)^−1^]] during the push off phase (Time of maximum force − Time at which force reach the body weight) [29,30]. The highest jump was used for further analysis (Intraclass Correlation Coefficients (ICC) = 0.91, 95% Confidence Intervals (CI; Lower = 0.90, Upper = 0.99, *n* = 13), coefficient of variation (CV) = 8.1%. 

#### 2.4.2. Leg Press Rate of Force Development

Fifteen minutes after the CMJs, leg press maximum isometric force and RFD were evaluated. Throwers were seated on a custom-made completely rigid leg press chair made of steel with rigid plywood boards and placed both their feet on the force platform (Applied Measurements Ltd. Co., Aldermaston, Berkshire, UK, WP800, 1000 kg weighting platform, 80 × 80 cm, sampling frequency 1 kHz), which was positioned perpendicular on a concrete laboratory wall. Knee angle was set at 120° and hip angle was set at 100° [7,8,13,30,31]. After three sub-maximal efforts, athletes were instructed to apply their maximum force as fast as possible for 3 s. Three maximum efforts with 3-min rest were performed while athletes were vocally encouraged to perform their maximum. Data from the force platform were recorded and analysed (Kyowa sensor interface PCD-320A; Kyowa Electronic Instruments Co., Ltd., Chofu, Tokyo, Japan) to calculate the maximum isometric force and the RFD from the force time curve. The signal was filtered using a secondary low pass Butterworth filter with a cutoff frequency of 20 Hz, automatically from the analysis software. The best performance according to RFD_100ms_ was used for statistical analysis. Rate of force development was calculated as the mean tangential slope of the force–time curve in specific time windows of 0–30, 0–50, 0–80, 0–100, 0–150, 0–200, and 0–250 ms, relative to the onset of contraction which was set at 2.5% of the difference between baseline and maximum force; e.g., RFD = DForce/DTime [1,32]. Early RFD includes the previously mentioned time points until the first 100 ms from the onset of a muscle contraction, while late RFD the time points over the first 100 ms from the onset of a muscle contraction [2,7,8,32,33]. Sequential RFD time windows were analyzed, including 30–50 ms, 50–80 ms, 80–100 ms, 100–150 ms, 150–200 ms, and 200–250 ms, while isometric force production expressed per percentage of maximum voluntary contraction (%MVC) in all time windows [(Fms/MVC)·100] was also calculated. MVC was calculated as the highest instantaneous force over the entire force-time curve force. Finally, RFD was expressed per lower body lean mass, per VL muscle thickness, and per maximum isometric force. The ICC for the leg press maximum isometric force, and RFD were calculated during the pilot study: ICC = 0.90 (95% CI: Lower = 0.86, Upper = 0.96), CV = 15%, and ICC = 0.92 (95% CI: Lower = 0.80, Upper = 0.98), CV = 12%, respectively (*n* = 13). 

#### 2.4.3. Leg Extension Rate of Force Development

Fifteen minutes after the isometric leg press test the leg extension test was performed. The athletes were positioned on a custom-made steel chair. Hip angle was set at 110° and initial knee joint angle was set at 70° (0° = full extension), [31,32]. Straps were used to ensure the stable position of the shoulders, hips, non-exercising leg, and the right angle of the exercising knee, as determined during the familiarization session [34]. Measurement was performed using a tension sensor (Applied Measurements Ltd., DBBE-1000 kg, Aldermaston, Berkshire, UK, sampling frequency 1000 Hz), which was positioned between the leg of extension and the immovable back of the chair attached with a metal ring. The distance between the ankle strap, where the tension sensor was attached and the center of the knee, was measured to the nearest mm to calculate the extension torque. Two submaximal efforts were performed with progressively increasing force. Then, three maximal efforts were allowed. Athletes were instructed to apply their maximum force as fast as possible and to sustain it for 3 s. Data from the tension sensor were recorded and analyzed (Kyowa Sensor Interface PCD-320A Kyowa Electronic Instruments Co., Ltd., Chofu, Tokyo, Japan) to calculate the maximum isometric torque and the RFD torque (N·m·s^−1^) from the force-time curve, as described in the previous paragraph, while the torque was corrected for the effect of gravity on the lower leg [32]. The best performance according to RFD_100ms_ was used for statistical analysis. The ICC for the leg extension maximum isometric force and RFD, were calculated during the pilot study and were: ICC = 0.98, (95% CI: Lower = 0.93, Upper = 0.99), CV = 12%, and ICC = 0.93, (95% CI: Lower = 0.79, Upper = 0.98), CV = 10%, respectively (*n* = 11). 

#### 2.4.4. Muscle Architecture

B-mode ultrasound images were obtained during the morning hours from vastus lateralis (VL) and medial gastrocnemius (GM) of the dominant leg using a 38-mm linear probe (Product model Z5, Shenzhen Mindray Bio-Medical Electronics Co., Ltd., Shenzhen, China). Extended-Field-Of-View mode was used to obtain panoramic images along the fascicle length, according to previous instructions [12,35]. For the assessment of VL architecture, participants laid supine with their knees fully extended and their muscles relaxed. A mark was drawn at 50% of the distance from the central palpable point of the greater trochanter to the lateral condyle of the femur [16]. For the measurement of GM architecture, participants lay prone with their knees fully extended, their feet hanging off the edge of the medical bed, and were asked to leave their feet rested and their muscles relaxed so that ankle angle was in a physiological position. Then the GM was marked at the proximal level of 30% of the distance between the popliteal crease and the center of the lateral malleolus [36]. A water-soluble gel was applied to the transducer to aid acoustic coupling and to reduce the needed pressure from the probe against the muscle. The transducer was placed longitudinal at femur or tibia, oriented in parallel to the muscle fascicles, and perpendicular to the skin. Due to individual differences, the transducer was sometimes aligned slightly diagonally to the longitudinal line of the muscle so that several fascicles could be easily delineated without interruption across the image. Based on this orientation, a dashed line (approximately 10 cm in length) was drawn forwards and backwards to the mark, to identify and capture the largest, continuous fascicle visualization. To obtain the muscle image, a continuous single view was taken by moving the probe along the marked, dashed line. Also, the mediolateral angle of the probe was changed throughout the experiment so that it remained perpendicular to the skin, which ensures the most reliable image acquisition for the measurement of the fascicle length [12]. Images were analysed for muscle thickness, fascicle angle, and fascicle length with image analysis software (Motic Images Plus 2.0, Motic, Hong Kong, China). Muscle thickness was defined as the mean of the distances between the superficial and deep aponeurosis measured at the ends of each panoramic image [26,27], fascicle angle as the angle of insertion of muscle fascicles into the deep aponeurosis, and fascicle length as the fascicular path between the insertion of the fascicle into the upper and deeper aponeurosis. The reliability for the measurement of muscle thickness (ICC = 0.976 [95% CI: 0.954–0.988], *p* = 0.001), fascicle angle (ICC = 0.862 [95% CI: 0.746–0.928], *p* = 0.001), and fascicle length (ICC = 0.834 [95% CI: 0.700–0.911], *p* = 0.001) were determined on two consecutive days by the same investigator (*n* = 36), during the pilot study.

#### 2.4.5. Body Composition Analysis

Τotal body scans were obtained with dual X-ray absorptiometry (model DPX-L; LUNAR Radiation, Madison, WI, USA). All of the measurements were analysed using the LUNAR radiation body composition program (LUNAR Radiation Body Composition Program). Analyses included fat mass, bone mineral density (BMD), and lean body mass (LBM) for total body, arms, trunk, and legs, according to manufacturer instructions. The ICC for body composition analysis was determined from two different researchers ICC = 0.98, (95% CI: Lower = 0.95, Upper = 0.99), CV = 9%, *n* = 13.

### 2.5. Statistics Procedures

All data are represented as mean ± SD. The comparisons between the two groups were also presented as percentage differences between them. One-way ANOVA was used to compare performance and biological variables between taekwondo athletes and track and field throwers. Effect sizes (*η*^2^) were also calculated (Palland, 2005). Pearson’s (*r*) product moment correlation coefficient was used to explore the relationships between variables. Standard multiple regression analysis was performed, including both groups of athletes. Because of the relative small sample size (*n* = 18) adjusted R squared was used for the interpretation of the multiple regression analysis results as the percentage explained variation [37]. Within subject’s variation and reliability was determined for all of the variables by calculating the confidence limits (95% CI), intraclass correlation coefficient (ICC), and coefficient of variation (CV), as described before [38]. Significance was accepted at *p* ≤ 0.05. All statistical analyses were performed using SPSS version 17.0 software (SPSS Inc., Chicago, IL, USA).

## 3. Results

Taekwondo athletes had lower body mass (46.8%, *p* = 0.000, *η*^2^ = 0.791), as well as total lean mass (35.1%, *p* = 0.000, *η*^2^ = 0.877) and bone mineral density (10.8%, *p* = 0.002, *η*^2^ = 0.473, Table 1) when compared to throwers. Also, taekwondo athletes had 14.6%, (*p* = 0.030, *η*^2^ = 0.254) lower CMJ height, lower CMJ power (69.8%, *p* = 0.000), lower CMJ power relative to body mass (12.8%, *p* = 0.015), lower CMJ maximum velocity (14.6%, *p* = 0.037, *η*^2^ = 0.258), lower leg press, and leg extension maximum isometric force/torque (63.7% and 63.8%, *η*^2^ = 0.643 and *η*^2^ = 0.765, respectively, *p* < 0.05, Table 2) when compared to throwers. Even when CMJ power was expressed relative to lean body mass, taekwondo athletes performed lower than throwers (28.4%, *p* = 0.015, *η*^2^ = 0.335, Table 1). Taekwondo athletes had lower leg press isometric force (19.4%, *p* = 0.007, *η*^2^ = 0.643) and leg extension torque (13.4%, *p* = 0.036, *η*^2^ = 0.261, Table 2) relative to total lean body mass as compared to throwers. Taekwondo athletes had significantly lower VL and GM muscle thickness (23.6% and 15.6%, *η*^2^ = 0.398 and *η*^2^ = 0.248, *p* < 0.05, respectively), as well as VL fascicle angle (33.6%, *p* = 0.001, *η*^2^ = 0.511), when compared to throwers. 

**Figure 1:**
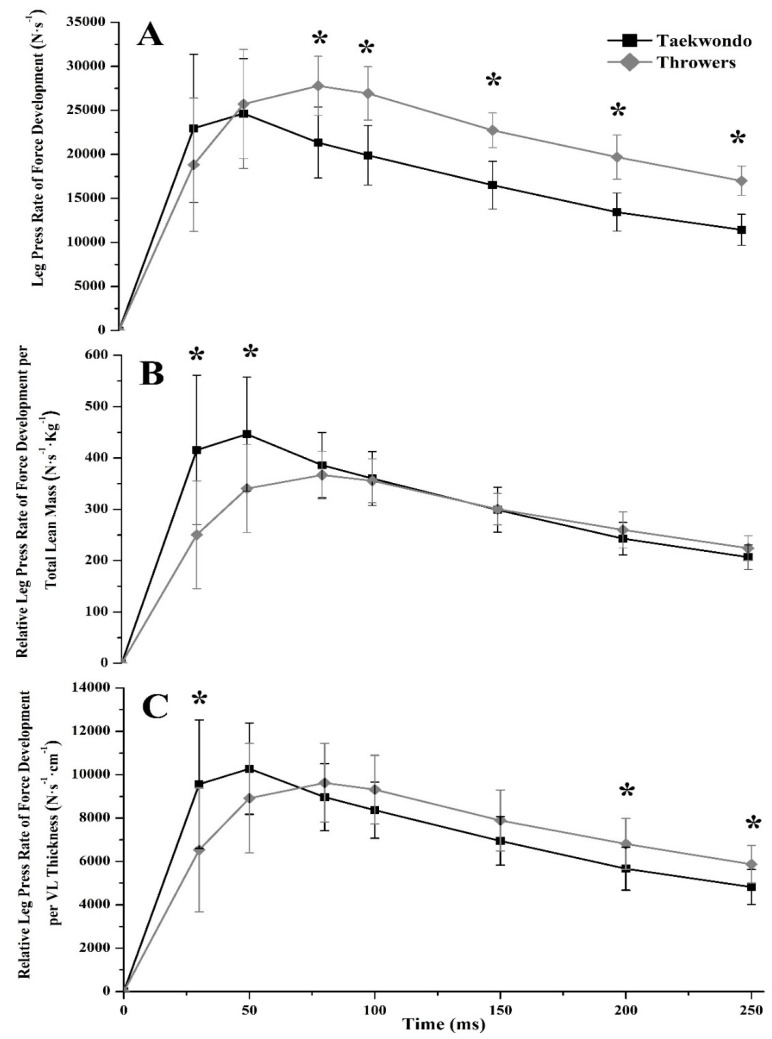
Leg press rate of force development (RFD) curves of taekwondo (black line) and thrower (grey line) athletes. (**A**) Leg press RFD curve in absolute values. Throwers had significantly higher RFD between 80–250 ms as compared to taekwondo athletes (* *p* < 0.05). (**B**) Leg press RFD curve relative to total lean mass. Taekwondo athletes had significantly higher RFD compared to throwers at 30 ms and 50 ms after contraction initiation (* *p* < 0.05). (**C**) Leg press RFD curve relative to vastus lateralis muscle thickness. Taekwondo athletes had significantly higher early RFD_30ms_ compared to throwers, while throwers had significantly higher late RFD_200–250ms_ (* *p* < 0.05) compared to taekwondo athletes.

**Figure 2:**
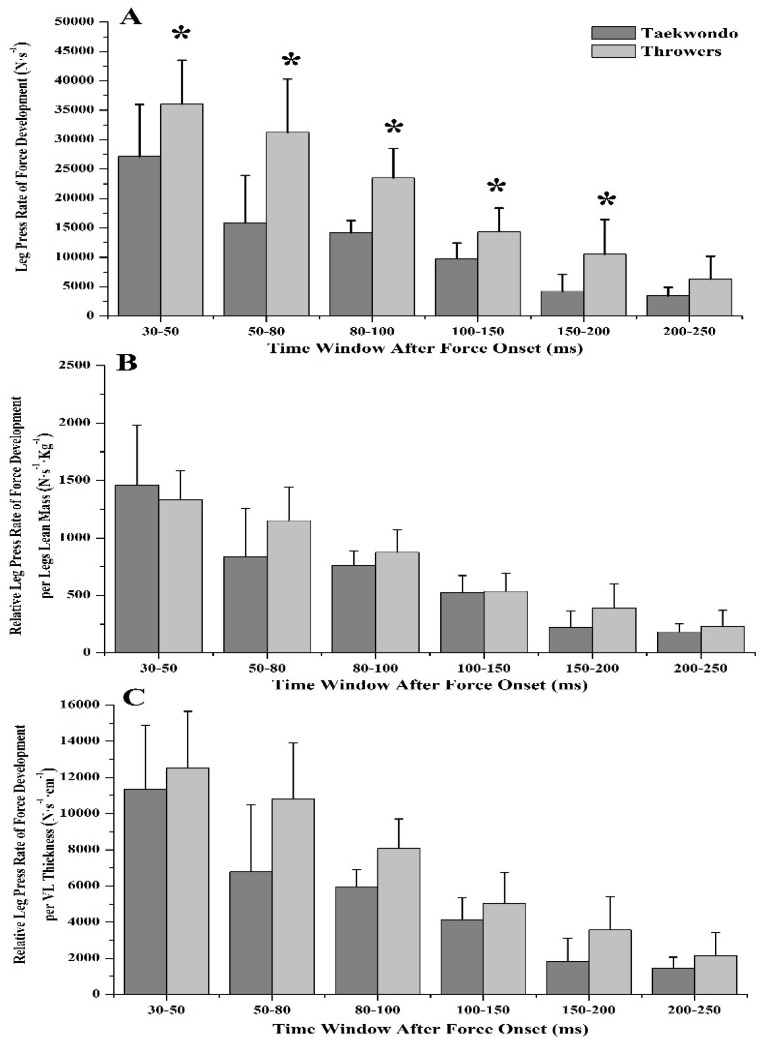
Leg press sequential rate of force development (SRFD) for taekwondo athletes (dark grey) and track and field throwers (light grey). (**A**) Leg press SRFD in absolute values. Track and field throwers had greater SRFD than taekwondo except during for the 200–259 ms time window where no difference exists. (**B**) Leg press SRFD relative to lower body lean mass. No significant difference was found between taekwondo and throwers. (**C**) Leg press SRFD relative to VL muscle thickness. No significant difference was found between taekwondo and throwers (* *p* < 0.05).

**Figure 3:**
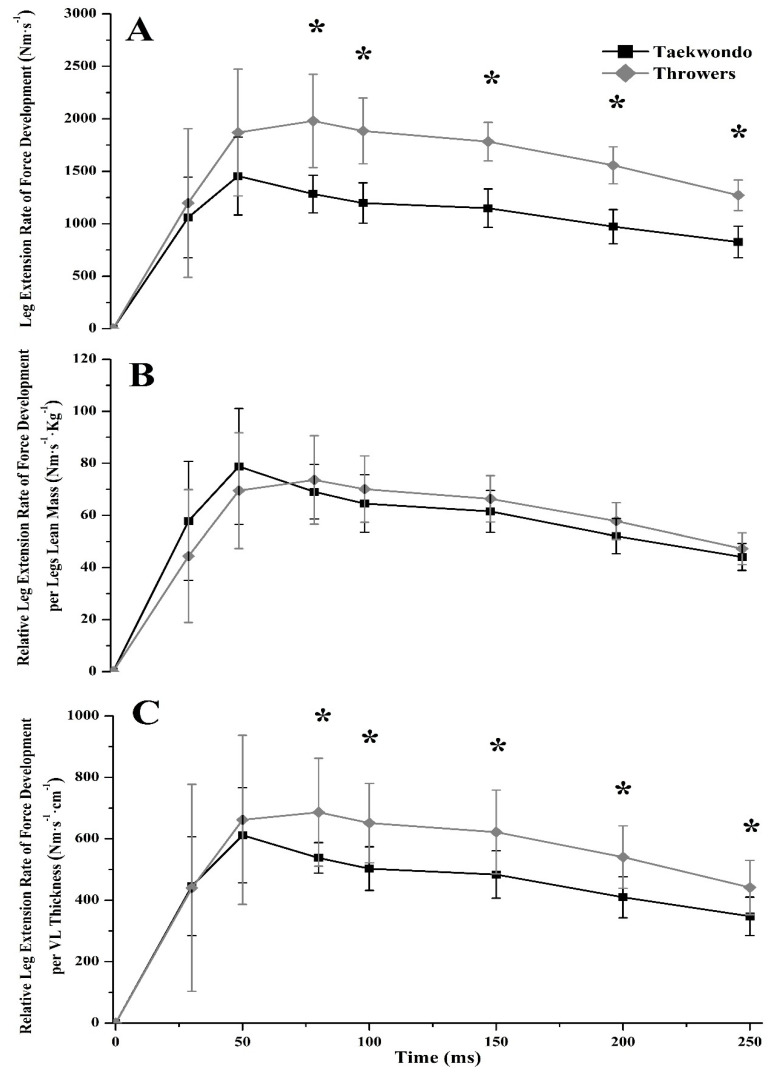
Leg extension rate of force development (RFD) curves calculated for taekwondo athletes (black line) and track and field throwers (grey line). (**A**) Leg extension RFD curve in absolute values. Throwers had significantly higher RFD between 80–250 ms compared to taekwondo athletes (* *p* < 0.05). (**B**) Leg extension RFD curve relative to legs lean mass. No significant difference was found between taekwondo and throwers. (**C**) Leg extension RFD curve relative to vastus lateralis muscle thickness. Throwers had significantly higher RFD between 80–250 ms as compared to taekwondo athletes (* *p* < 0.05).

**Figure 4:**
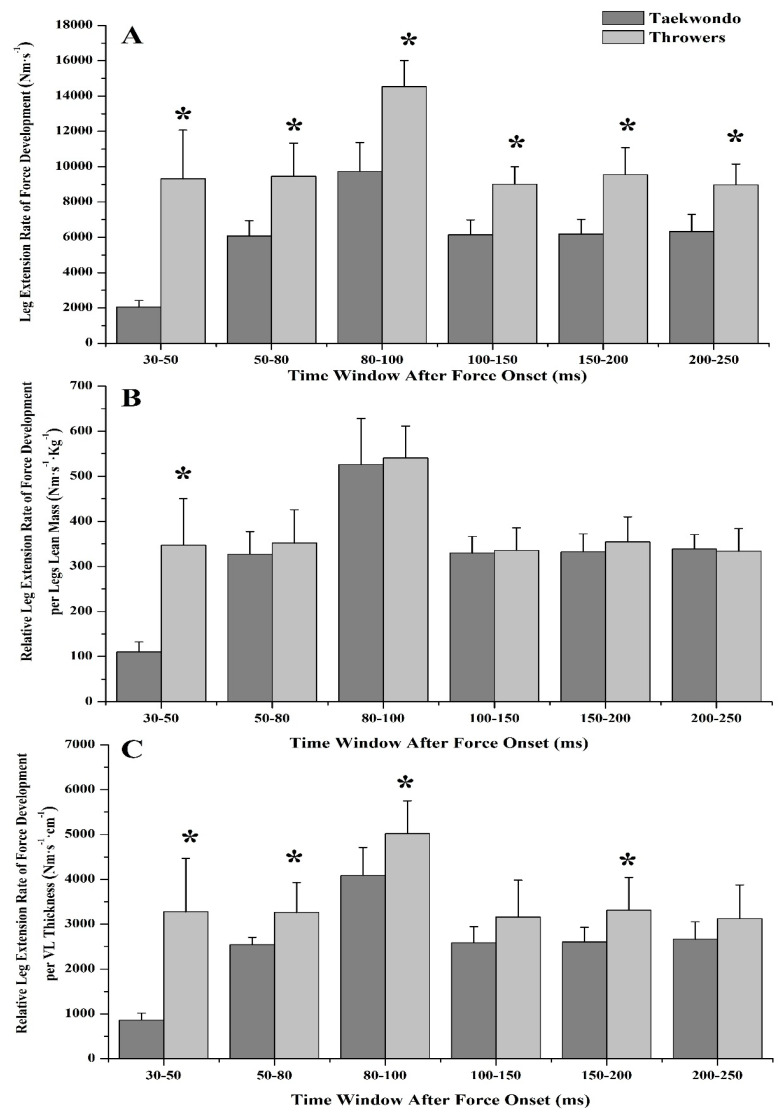
Leg extension sequential rate of force development (SRFD) for taekwondo athletes (dark grey) and track and field throwers (light grey). (**A**) Leg extension SRFD in absolute values. Track and field throwers had greater SRFD than taekwondo athletes in all time windows. (**B**) Leg extension SRFD relative to lower body lean mass. Significant difference was found between during 30–50 ms for the taekwondo athletes compared to throwers. (**C**) Leg extension SRFD relative to VL muscle thickness. Significant difference was found during 30–50, 50–80, 80–100, and 150–200 ms for throwers in comparison to taekwondo (* *p* < 0.05).

No significant differences were found between groups in RFD until the first 50 ms of the force/torque-time curve, either in leg press or in leg extension (*η*^2^ = 0.009–0.158, Table 2). Yet, taekwondo athletes had lower leg press RFD and lower sequential RFD later in the force/torque-time curve (80 ms = 30.2%, 100 ms = 35.3%, 150 ms = 37.7%, 200 ms = 46.4%, 250 ms = 48.6%, *p* < 0.05, *η*^2^ = 0.440–0.616, Figure 1A and Figure 2A), as well as in leg extension (80 ms = 47.8%, 100 ms = 51.0%, 150 ms = 49.6%, 200 ms = 51.5%, 250 ms = 48.7%, *p* < 0.05, *η*^2^ = 0.532–0.771, Figure 3A and Figure 4A) as compared to throwers. 

Taekwondo athletes had higher leg press RFD at 30 ms and 50 ms relative to their lean body mass (30 ms = 39.8%, *p* = 0.016, *η*^2^ = 0.329, 50 ms = 23.7%, *p* = 0.043, *η*^2^ = 0.246, Figure 1B) when compared to throwers. Also, when RFD was expressed relative to VL muscle thickness, taekwondo athletes had higher leg press RFD at 30 ms (difference between groups for RFD_30ms_ = 31.7%, *p* = 0.049, *η*^2^ = 0.235) as compared to throwers, while throwers had higher leg press RFD at 200 and 250 ms (difference between groups for RFD_200ms_ = 20.1%, *p* = 0.048, *η*^2^ = 0.237, and RFD_250ms_ = 21.7%, *p* = 0.021, *η*^2^ = 0.307, Figure 1C) when compared to taekwondo athletes. Additionally, no significant difference was observed for leg press sequential RFD relative to lean mass or VL thickness between taekwondo and throwers (Figure 2B,C). Leg extension sequential RFD was lower for taekwondo athletes at 30–50 ms relative to lower body lean mass as compared to throwers (68.2%, *p* = 0.000, *η*^2^ = 0.727), while leg extension sequential RFD expressed per VL thickness was lower for taekwondo at 30–50, 50–80, 80–100, and 150–200 ms, respectively (difference between groups for RFD_30–50ms_ = 73.7%, *p* = 0.000, *η*^2^ = 0.684, RFD_50–80ms_ = 22.1%, *p* = 0.010, *η*^2^ = 0.368, RFD_80–100ms_ = 18.5%, *p* = 0.013, *η*^2^ = 0.343, RFD_150–200ms_ = 21.4%, *p* = 0.024, *η*^2^ = 0.297, Figure 4B,C).

**Figure 5:**
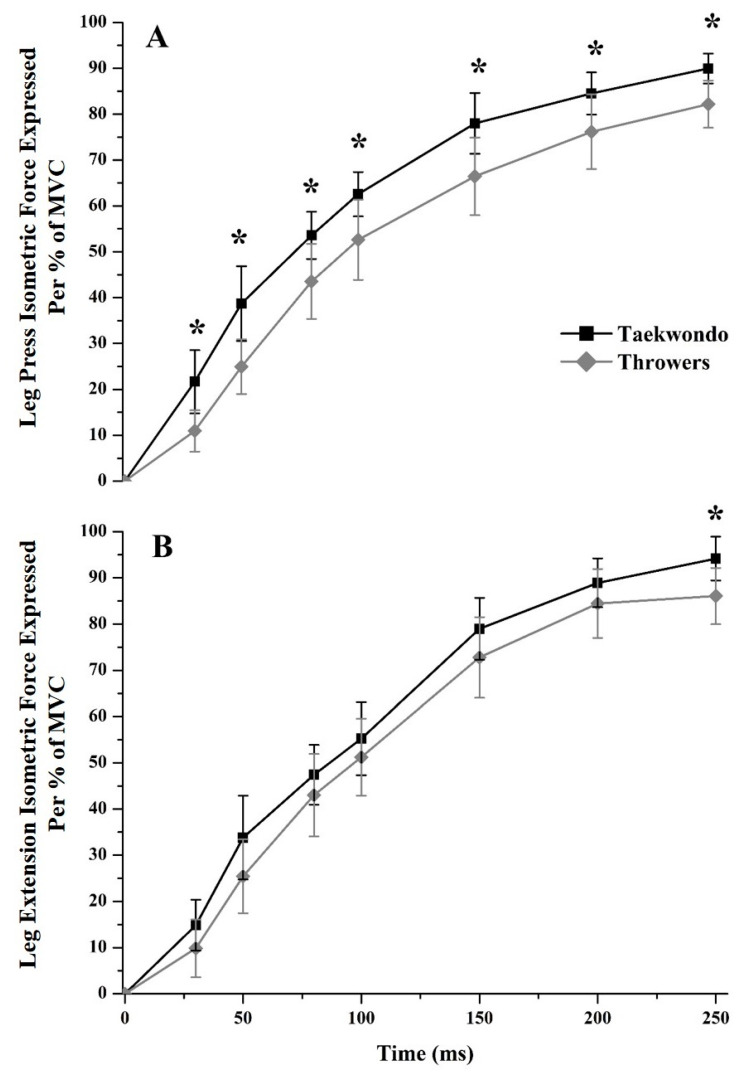
Leg press and leg extension isometric force production (IFP) expressed per maximum volunteer contraction (%MVC) for taekwondo athletes (black line) and track and field throwers (grey line). (**A**) Taekwondo had higher IFP %MVC than track and field throwers in all time moments for leg press IFP %MVC. (**B**) Taekwondo had higher leg extension IFP %MVC only during 250 ms as compared to thrower athletes (* *p* < 0.05).

Taekwondo athletes had higher leg press isometric force production (IFP) expressed per percentage of maximum volunteer contraction (%MVC) than throwers at all time windows (30 ms = 98%, *p* = 0.002, *η*^2^ = 0.497, 50 ms = 55%, *p* = 0.001, *η*^2^ = 0.519, 80 ms = 23%, *p* = 0.009, *η*^2^ = 0.371, 100 ms = 19%, *p* = 0.012, *η*^2^ = 0.350, 150 ms = 17%, *p* = 0.007, *η*^2^ = 0.391, 200 ms = 11%, *p* = 0.022, *η*^2^ = 0.304, and 250 ms = 9%, *p* = 0.002, *η*^2^ = 0.473, Figure 5A). Additionally, in leg extension taekwondo athletes had higher IFP per %MVC only at 250 ms (250 ms = 9%, *p* = 0.008, *η*^2^ = 0.380, Figure 5B). 

**Figure 6:**
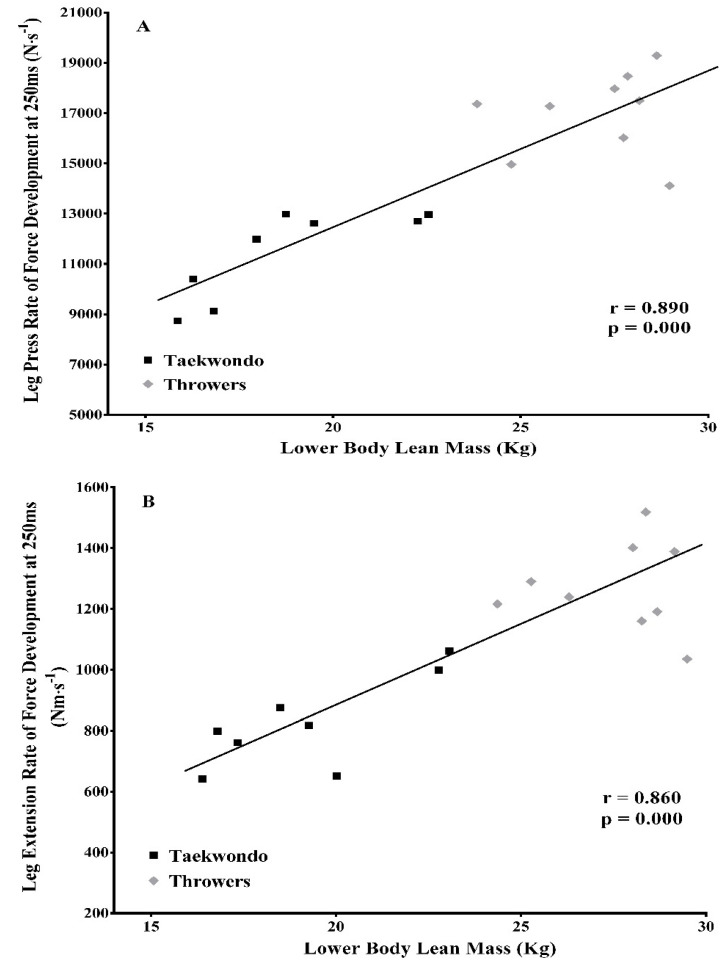
(**A**) Significant correlation between lower body lean mass and leg press RFD for taekwondo athletes (black dots) and track and field throwers (grey dots). (**B**) Significant correlation between lower body lean mass and leg extension RFD for taekwondo athletes (black dots) and track and field throwers (grey dots).

When all athletes were considered as a group, lean mass of the lower extremities was significantly correlated with leg press RFD_250ms_ (*r* = 0.89, *p* = 0.000, Figure 6A) and leg extension RFD_250ms_ (*r* = 0.86, *p* = 0.000, Figure 6B). In taekwondo athletes, lean mass of the lower extremities was significantly correlated with average RFD during the CMJ (*r* = 0.710, *p* = 0.041) as well as leg press RFD_100ms_ (*r* = 0.680, *p* = 0.047), RFD_200ms_ (*r* = 0.730, *p* = 0.035), RFD_250ms_ (*r* = 0.810, *p* = 0.016), and leg press maximum isometric force (*r* = 0.83, *p* = 0.008). Also, in taekwondo athletes, lean mass of the legs was significantly correlated with leg extension RFD_250ms_ (*r* = 0.760, *p* = 0.026) and leg extension maximum isometric torque (*r* = 0.800, *p* = 0.011). In contrast, no significant correlations were observed between lean body mass and performance for the throwers. 

Vastus lateralis muscle thickness was significantly correlated with leg extension RFD_80ms_ (*r* = 0.780, *p* = 0.018) while GM muscle thickness was correlated with leg press maximum isometric force (*r* = 0.670, *p* = 0.049), in taekwondo athletes. VL fascicle length was significantly correlated with leg press RFD_250ms_ (*r* = 0.67, *p* = 0.05) in throwers. The linear combination of VL muscle thickness and VL fascicle length could significantly predict the power performance in CMJ and the RFD between 80–250 ms, as well as maximum isometric force/torque for both leg press and leg extension (*N* = 18, Table 3).

## 4. Discussion

The aim of the study was to describe the relationship between lower-body lean mass and RFD and to compare lower-body RFD in taekwondo athletes and track and field throwers, the latter having higher lean body mass when compared to taekwondo athletes. The main findings of the present study were that (1) lower body lean mass was significantly correlated with RFD in power-trained athletes having large lean body mass differences, (2) taekwondo athletes had greater early RFD when this was expressed relative to total lean mass or vastus lateralis muscle thickness, as compared to track and field throwers, and (3) throwers performed better in late RFD when compared to taekwondo athletes both in absolute values as well as relative to total lean mass or vastus lateralis thickness. Unfortunately, in the current study neither the neural components nor muscle fiber composition were assessed, therefore the contribution of these components to the early phase of force rise was not explored. However, it is reasonable to assume that longitudinal taekwondo training adaptations induced in response to fast muscle actions performed with relative low external resistance (e.g., body mass, [15]) might have resulted in increased early RFD. Taekwondo is a combat sport that depends on both agility-speed and power production. Anecdotal data from coaches suggests that during competition athletes must use their lower limbs as fast as possible to achieve a successful attack while often earning a point with a reflex-respond kick to the opponent’s offence, which is a teaching strategy, an action that it has to be completed instantaneously. According to these, it seems that early RFD might be of significance for these athletes, while their lower legs’ lean mass could, at least partly, explain their high early RFD relative to their musculature in comparison to track and field throwers. 

Conversely, the delivery phase in track and field throws ranges between 150–250 ms [3,39,40,41]. Chronic exercise training adaptations may have increased the late RFD ability of these athletes rendering them to perform better relative to their musculature as compared to taekwondo athletes, as found in the present study. Throwers had higher maximum isometric force relative to lean body mass which suggests an improved recruitment of their muscle tissue after the initial milliseconds of a maximum contraction. This might be the result of chronic resistance training adaptations in these athletes as opposed to the taekwondo athletes who do not regularly use high external resistances during training [15]. However, given that both taekwondo athletes and throwers have similar critical time periods to develop their maximum force or power, it would be of interesting, future studies to investigate if a training program, similar to that of the throwers, could increase the performance of Taekwondo athletes.

When RFD was expressed relative to total lean mass and legs lean mass, taekwondo athletes had higher early RFD than throwers in leg press but not in leg extension (no significant difference between groups for the leg extension). Although leg extension is commonly used to assess RFD, it is not regularly performed during training by either taekwondo athletes or track and field throwers. Thus, specific neural adaptations may not have developed for both groups for the leg extension, which might explain these results. In contrast, the multi-articular leg press exercise is a movement regularly performed by athletes either as specific leg press or in a broader sense in jumping, squatting, etc. Neural adaptations may be more applicable in leg press, which might explain the closer link with RFD relative to muscle mass/thickness. 

When all the athletes were considered together, lean body mass was significantly correlated with late RFD. The close link between muscle mass and late RFD has been predicted in earlier studies where training-induced increases in late RFD correlated with chronic increases in muscle mass [1,10,11,13]. Similar correlations were also found for the taekwondo athletes but not for the throwers in the current study. Significant correlations between lean body mass and late RFD_100–250ms_ have been recently reported in young well-trained track and field throwers [14]. However, the athletes that participated in that previous study had lower muscle mass (approximately 57.1 ± 13.2 kg, total lean mass) when compared to the athletes that participated in the current study (total lean mass = 75.9 ± 3.4 kg). This may suggest that the impact of muscle mass on late RFD may not be linear when large muscle masses have been accomplished. 

The role of muscle architecture in power performance and the RFD has been debated. Some studies have shown a link between fascicle length or fascicle angle and muscle power [14,21,28,42,43], while other studies failed to reveal similar results [28,44,45]. This discrepancy might be due to the differential effect of muscle architecture in the early and late RFD. In a recent study, early RFD during jumping (10–30 ms) has been linked with the gastrocnemius fascicle length [46]. In the present study, neither vastus lateralis nor was gastrocnemius architecture correlated with early RFD. However, vastus lateralis fascicle length was significantly correlated with leg press late RFD in throwers. Moreover, late RFD could be predicted from the linear combination of vastus lateralis thickness and fascicle length when all athletes were considered together. This suggests that in power-trained individuals the combination of muscle hypertrophy (thickness) and the number of sarcomeres in series (fascicle length) might be vital for power production. Similar results were recently reported in a group of young track and field throwers where the linear combination of vastus lateralis muscle thickness and fascicle length could predict 53% of the shot put performance [14]. Future studies should address this intriguing issue more systematically, perhaps in a larger number of power-trained individuals. Finally, in the present study, RFD was evaluated in testing positions that were different to the ultrasound collection position. Recent study report that performing ultrasonography evaluation of vastus lateris architecture with athletes in a lying position or in a different position when compared to those that they have during strength and power evaluations, does not allow for us to have a precise view of muscle configuration that is present during performance evaluations, and this may have some impact on the correlations between strength/power performances and architecture parameters [18]. Unfortunately, the present study performed prior to this report. Thus, the lying position during the architecture evaluation of vastus lateralis, may have some impact on the results of the present study, providing lower or non-significant correlations as compared to those that may be found if the evaluations were performed in a position that is close to those of the performance evaluations [18]. 

Unfortunately, it was not possible to evaluate the electromyographic activity in this study. This might have provided further insight into the current results, especially about the neural input in the early phase of the RFD curve. Another limitation of this study was the lack of information regarding the muscle fiber composition of the athletes. Previous studies have shown a higher percentage of vastus lateralis area that is covered with type II muscle fibers in track and field throwers [47,48]. Although similar data might be expected for the taekwondo athletes, to our knowledge there are no such experimental data. However, a possible difference between the percentage of the vastus lateralis area that is covered with type II muscle fibers between the two groups of athletes in the current study might explain the differences that are found in early and late RFD relative to lean mass or muscle thickness. Furthermore, the relative small number of athletes in this study as well as the testing procedures (e.g., lack of warm-up activities prior the RFD testing) may pose limitations regarding the generalization of the results. 

## 5. Conclusions

In conclusion, the current results reveal a significant correlation between lower body lean mass and RFD in power-trained athletes having large inter-individual differences in lean body mass. The track and field throwers are stronger than taekwondo athletes and they have more power in countermovement jumping. When the RFD is expressed relative to lean body mass, taekwondo athletes perform better that the throwers in the initial 30–50 ms after the initiation of an explosive contraction, which might be related to a higher neural input. In contrast, track and field throwers perform better relative to their lean muscle mass in the late part of the RFD curve. 

In practice, the current results suggest that taekwondo athletes and coaches should regularly measure the lower body RFD, especially the early part of the force rise (<100 ms), as well as the lean body mass to evaluate the lower body power adaptations induced with training. The importance of the early RFD in taekwondo athletes revealed in this study suggests that these athletes should implement explosive actions with moderate to low external resistance (e.g., drop jumps) in their training. In contrast, in sports demanding explosive movements against higher external loads, such as the track and field throws, training may mainly focus on increasing the later part of the force rise of the force-time curve (>150 ms) and laboratory testing of the RFD may also focus on this part of the curve. For these athletes, development of lean body mass seems to be an important factor for the late RFD. 

## Figures and Tables

**Table 1:** Body composition, jumping performance, and muscle architecture in taekwondo athletes (*n* = 9) and track and field throwers (*n* = 9).

Evaluation	Parameter	Taekwondo	Throwers	*p*	*η* ^2^
Body composition	Body height (cm)	174.3 ± 4.9	183.3 ± 4.9	0.002	0.486
	Body mass (kg)	66.3 ± 7.8	99.3 ± 9.9	0.000	0.791
	LBM total (kg)	55.2 ± 4.9	75.9 ± 3.4	0.000	0.877
	LBM arms (kg)	7.8 ± 1.1	9.6 ± 0.8	0.002	0.509
	LBM trunk (kg)	25.0 ± 2.0	35.6 ± 2.0	0.000	0.886
	LBM legs (kg)	18.7 ± 2.8	27.0 ± 1.8	0.000	0.789
	BMD total (g·cm^2^)	1.3 ± 0.09	1.5 ± 0.08	0.002	0.473
	Body fat (%)	10.9 ± 7.1	17.3 ± 5.0	0.053	0.241
CMJ	Jump height (cm)	39.9 ± 5.9	45.8 ± 5.5	0.037	0.254
	Max power (W)	918.4 ± 149.1	1578.6 ± 280.2	0.000	0.702
	Max power/LBM (W/kg)	16.7 ± 2.5	20.8 ± 3.5	0.015	0.335
	Max velocity (m·s^−1^)	3.3 ± 0.5	3.8 ± 0.5	0.037	0.258
	ARFD (N·s^−1^)	546.1 ± 168.9	649.6 ± 218.2	0.296	0.072
VL architecture	Thickness (cm)	2.4 ± 0.2	3.0 ± 0.5	0.007	0.398
	Fascicle angle (°)	17.0 ± 2.5	22.7 ± 3.3	0.001	0.511
	Fascicle length (cm)	8.4 ± 0.4	8.4 ± 0.8	0.888	0.001
GM architecture	Thickness (cm)	1.9 ± 0.4	2.3 ± 0.2	0.042	0.248
	Fascicle angle (°)	22.2 ± 4.6	25.7 ± 4.4	0.134	0.143
	Fascicle length (cm)	6.2 ± 0.7	5.9 ± 0.9	0.521	0.033

LBM = Lean body mass, BMD = Bone mineral density, CMJ = Counter movement jump, ARFD = Average rate of force development, VL = Vastus lateralis, GM = Medial gastrocnemius.

**Table 2:** Leg press and leg extension rate of force development, maximum isometric force/torque in taekwondo athletes and track and field throwers.

Parameter		Taekwondo	Throwers	*p*	*η* ^2^
**Leg Press**					
MIF (kg)		349.6 ± 75.5	529.9 ± 67.5	0.000	0.643
MIF/Legs Lean Mass (kg·kg^−1^)		5.8 ± 0.5	6.9 ± 0.9	0.007	0.395
Rate of Force Development (N·s^−1^)	30 ms	22,967.6 ± 8403.7	18,827.9 ± 7567.6	0.717	0.009
	50 ms	24,635.3 ± 6243.5	25,715.2 ± 6206.4	0.267	0.082
	80 ms	21,341.3 ± 4035.4	27,784.1 ± 3362.9	0.004	0.440
	100 ms	19,903.2 ± 3388.9	26,929.5 ± 3036.1	0.003	0.457
	150 ms	16,512.6 ± 2703.1	22,735.9 ± 1973.4	0.001	0.519
	200 ms	13,441.5 ± 2171.2	19,683.7 ± 2509.5	0.001	0.506
	250 ms	11,440.9 ± 1761.9	16,996.5 ± 1668.7	0.000	0.616
Sequential Rate of Force Development (N·s^−1^)	30–50 ms	27,136.9 ± 8846.5	36,046.3 ± 7473.6	0.040	0.252
	50–80 ms	15,851.3 ± 8083.4	31,232.1 ± 9088.6	0.002	0.472
	80–100 ms	14,150.9 ± 2103.6	23,511.3 ± 4994.8	0.000	0.617
	100–150 ms	9731.5 ± 2688.3	14,348.8 ± 3996.6	0.015	0.336
	150–200 ms	4228.1 ± 2861.2	10,527.2 ± 5904.6	0.015	0.333
	200–250 ms	3438.4 ± 1433.5	6247.8 ± 3930.5	0.076	0.195
**Leg Extension**					
MIT (N·m)		218.6 ± 34.5	371.2 ± 52.3	0.000	0.765
MIT/Legs Lean Mass (Nm·Kg^−1^)		2.9 ± 0.2	3.4 ± 0.4	0.036	0.261
Rate of Force Development (Nm·s^−1^)	30 ms	1059.7 ± 384.3	1196.9 ± 707.6	0.613	0.016
	50 ms	1454.5 ± 370.2	1869.5 ± 604.2	0.114	0.158
	80 ms	1283.7 ± 179.3	1979.6 ± 444.5	0.001	0.532
	100 ms	1197.8 ± 191.8	1885.1 ± 313.8	0.000	0.657
	150 ms	1148.9 ± 182.8	1782.8 ± 184.3	0.000	0.771
	200 ms	972.6 ± 161.9	1557.2 ± 176.9	0.000	0.763
	250 ms	825.9 ± 150.1	1271.4 ± 146.1	0.000	0.719
Sequential Rate of Force Development (Nm·s^−^^1^)	30–50 ms	2046.7 ± 380.3	9319.3 ± 2752.4	0.000	0.784
	50–80 ms	6080.7 ± 870.4	9457.4 ± 1873.7	0.000	0.591
	80–100 ms	9732.9 ± 1642.8	14,536.2 ± 1475.2	0.000	0.729
	100–150 ms	6144.4 ± 840.3	9007.2 ± 992.3	0.000	0.730
	150–200 ms	6187.2 ± 836.6	9554.4 ± 1532.6	0.000	0.669
	200–250 ms	6332.5 ± 966.4	8975.1 ± 1171.9	0.000	0.628

RFD = Rate of force development. MIF = Maximum isometric force. MIT = Maximum isometric torque.

**Table 3:** Results from multiple linear regression analysis for both groups of taekwondo and thrower athletes (*N* = 18).

Dependent	Independent	ANOVA	Variable Explained (%)	β	Sig.
CMJ Power	VL Thickness	*F* = 5.746 *p* = 0.015	37.2	0.850	0.004
	VL Fascicle Length			−0.573	0.039
LPRFD_80ms_	VL Thickness	*F* = 10.458 *p* = 0.002	54.2	0.982	0.000
	VL Fascicle Length			−0.588	0.016
LPRFD_100ms_	VL Thickness	*F* = 11.911 *p* = 0.001	57.7	1.000	0.000
	VL Fascicle Length			−0.522	0.024
LPRFD_150ms_	VL Thickness	*F* = 9.847 *p* = 0.002	52.5	0.964	0.001
	VL Fascicle Length			−0.509	0.035
LPRFD_200ms_	VL Thickness	*F* = 7.581 *p* = 0.006	45.1	0.895	0.002
	VL Fascicle Length			−0.401	0.110
LPRFD_250ms_	VL Thickness	*F* = 10.891 *p* = 0.001	55.3	0.972	0.000
	VL Fascicle Length			−0.447	0.054
LPRFD_MIF_	VL Thickness	*F* = 12.517 *p* = 0.001	59.0	0.985	0.000
	VL Fascicle Length			−0.410	0.063
LERFD_80ms_	VL Thickness	*F* = 4.305 *p* = 0.035	29.2	0.765	0.012
	VL Fascicle Length			−0.338	0.226
LERFD_100ms_	VL Thickness	*F* = 7.422 *p* = 0.006	44.5	0.884	0.002
	VL Fascicle Length			−0.372	0.138
LERFD_150ms_	VL Thickness	*F* = 7.224 *p* = 0.007	43.8	0.904	0.002
	VL Fascicle Length			−0.541	0.039
LERFD_200ms_	VL Thickness	*F* = 9.073 *p* = 0.003	50.2	0.950	0.001
	VL Fascicle Length			−0.515	0.037
LERFD_250ms_	VL Thickness	*F* = 7.958 *p* = 0.005	46.5	0.920	0.001
	VL Fascicle Length			−0.488	0.054
LERFD_MIF_	VL Thickness	*F* = 9.069 *p* = 0.003	50.2	0.936	0.001
	VL Fascicle Length			−0.434	0.073

VL = Vastus lateralis, CMJ = Counter movement jump, LPRFD = Leg press rate of force development, LERFD = Leg extension rate of force development, MIF = Maximum isometric force, β = Contribution of VL thickness and fascicle length to the multiple standard regression.
